# MitoQ Blunts Mitochondrial and Renal Damage during Cold Preservation of Porcine Kidneys

**DOI:** 10.1371/journal.pone.0048590

**Published:** 2012-11-06

**Authors:** Nirmala Parajuli, Lia H. Campbell, Akira Marine, Kelvin G. M. Brockbank, Lee Ann MacMillan-Crow

**Affiliations:** 1 Department of Pharmacology and Toxicology, University of Arkansas for Medical Sciences, Little Rock, Arkansas, United States of America; 2 Cell & Tissue Systems, Inc., North Charleston, South Carolina, United States of America; 3 Institute for Bioengineering and Bioscience, Georgia Institute of Technology, Atlanta, Georgia, United States of America; 4 Department of Regenerative Medicine and Cell Biology, Medical University of South Carolina, South Carolina, United States of America; National Institutes of Health, United States of America

## Abstract

Cold preservation has greatly facilitated the use of cadaveric kidneys for transplantation but damage occurs during the preservation episode. It is well established that oxidant production increases during cold renal preservation and mitochondria are a key target for injury. Our laboratory has demonstrated that cold storage of renal cells and rat kidneys leads to increased mitochondrial superoxide levels and mitochondrial electron transport chain damage, and that addition of Mitoquinone (MitoQ) to the preservation solutions blunted this injury. In order to better translate animal studies, the inclusion of large animal models is necessary to develop safe preclinical protocols. Therefore, we tested the hypothesis that addition of MitoQ to cold storage solution preserves mitochondrial function by decreasing oxidative stress, leading to less renal tubular damage during cold preservation of porcine kidneys employing a standard criteria donor model. Results showed that cold storage significantly induced oxidative stress (nitrotyrosine), renal tubular damage, and cell death. Using High Resolution Respirometry and fresh porcine kidney biopsies to assess mitochondrial function we showed that MitoQ significantly improved complex II/III respiration of the electron transport chain following 24 hours of cold storage. In addition, MitoQ blunted oxidative stress, renal tubular damage, and cell death after 48 hours. These results suggested that MitoQ decreased oxidative stress, tubular damage and cell death by improving mitochondrial function during cold storage. Therefore this compound should be considered as an integral part of organ preservation solution prior to transplantation.

## Introduction

Renal transplantation is the treatment of choice for end stage renal disease (ESRD), because it increases patient survival and quality of life, and reduces medical costs for ESRD patients [1]. Brain death and cardiac death donors (both termed deceased donors) are the major sources of donor kidneys used in transplantation. These kidneys are routinely flushed with and preserved in cold storage solution to prolong viability while being matched for recipients [2].

Static cold storage is a convenient and fairly inexpensive method for renal allograft preservation [3], [4]. Short-term cold storage reduces cellular oxygen demand, but prolonged storage can cause extensive renal damage within the tubular and medullar compartments as well as marked mitochondrial damage resulting in reduced graft function and survival [5]–[7]. This could be due to renal cell damage mediated by high amounts of oxidants generated by the mitochondria, especially superoxide [8]–[11]. Currently, there are few specific therapies or approaches to reduce oxidative stress mediated cellular damage prior to transplantation. One growing strategy, however, is to counter the detrimental effects of ischemia, mediated by mitochondrial superoxide, as a consequence of prolonged cold storage, thereby improving graft survival function following transplantation.

Mitoquinone or MitoQ™ is a mitochondrial targeted antioxidant compound and has an ubiquinol (antioxidant) moiety on one end and a triphenylphosphonium (charged lipophilic cation that targets mitochondria) moiety on the other end [12]. MitoQ has been shown to modulate mitochondrial oxidant formation, which has numerous downstream effects that could be involved with its protection in a variety of pathologies including ischemia/reperfusion (cardiac [13], hepatic [14]), sepsis [15], diabetes [16], [17], cisplatin-induced nephropathy [18], and chronic alcohol-induced liver disease [19]. Our earlier report showed that the addition of MitoQ to a cold storage solution partially protected renal tubular cells and rat kidneys against cold storage mediated injury [20]. Furthermore, this report demonstrated that MitoQ minimizes the impact of oxidative stress in the cells by lowering steady state superoxide levels and improving electron transport chain (ETC) activity.

Experimental animal models play a crucial role in all stages of developing future clinical strategies and therapeutic interventions for human health and diseases. Large animal models are particularly necessary to develop safe preclinical protocols that are directly transferable to human subjects due to similarities in anatomical structure, size and physiology as well as disease progression. The pig is considered to be an ideal large animal model for human disease research [21]. Using porcine kidney as a renal model, we tested the hypothesis that addition of MitoQ to the cold storage solution would decrease mitochondrial superoxide, as well as preserve mitochondrial function; and that both effects would lead to less renal tubular damage during cold preservation. The results show that MitoQ blunted the cold-storage mediated oxidative stress and tubular damage, and preserved mitochondrial function by partially stabilizing respiration.

## Methods

### Animals

A standard criteria donor model was employed in which pig kidneys were explanted approximately 10 minutes after cessation of heart beat and brain death. Kidneys were obtained from 10 male farm pigs (25–30 kg, Hambone Farms, SC) donating other abdominal organs in IACUC approved studies. At the conclusion of IACUC approved liver or pancreas organ harvesting procedures, the pigs were euthanized by exsanguination under anesthesia according to the latest guidelines from the American Veterinary Medical Association Panel on Euthanasia (AVMA). Animal care and handling complied with the “Principles of Laboratory Animal Care” as formulated by the National Society for Medical Research and the “Guide for the Care and Use of Laboratory Animals” published by the National Research Council (National Academy press, 1996).

The animals were weighed and pre-anesthetized with a mixture of ketamine (22 mg/kg), acepromazine (1.1 mg/kg) and atropine (0.05 mg/kg) given IM. After establishing an ECG, the pigs were intubated, and placed on isoflurane anesthesia at 1.5–2%. The pigs were anticoagulated with heparin (400 IU/kg, IV). A midline incision was made from the top of the sternum to the pubis. The chest was opened to allow access to clamp off the dorsal aorta and the inferior vena cava in the thorax. After cannulation of the abdominal dorsal aorta the inferior vena cava was cut above the diaphragm. The abdominal organs were then perfused with 4L of cold Lactated Ringers followed by 1L cold Belzer’s solution also known as UW solution (SPS-1, Organ Recovery Systems, Itasca, IL). The kidneys were excised (with the renal artery, vein and ureter attached) and immersed in cold Belzer’s solution, placed on ice and moved to the back table for renal cannulation.

### Cold Storage of Kidney

One of each pair of kidneys was allocated as an untreated control and the other experimental kidney to the MitoQ (100 µM) treatment group. 10–20 minutes after cessation of heartbeat, cannulation and time zero biopsy collection, the kidneys were flushed with cold Belzer’s solution ±100 µM MitoQ. The flush was performed at low pressure (20–40 mmHg) until the effluent was clear and residual blood eliminated. The kidneys were placed in sterile plastic bags with the remainder of 1L of cold Belzer’s solution ±100 µM MitoQ, respectively, and stored on ice during transportation to the research laboratory. The organs were then shipped in preservation solution (± MitoQ) on ice by express courier to the University of Arkansas for Medical Sciences (UAMS) for analysis. This type of organ transportation mimics that which occurs clinically with most human kidney donations. Biopsy specimens were then taken (at UAMS) at 24 h and 48 h of cold storage for histochemical and High Resolution Respirometry (HRR) analyses.

#### Cold storage group

Right kidneys were flushed with cold Belzer’s solution and stored in cold Belzer’s solution at 4°C for 24 and 48 h (n = 5).

#### Cold storage + MitoQ (100 µM) group

Left kidneys were flushed with cold Belzer’s solution and stored in cold Belzer’s solution +100 µM MitoQ at 4°C for 24 and 48 h (n = 5).

### Kidney Morphology Based on PAS Staining

Renal sections were assessed for tissue injury using the Periodic Acid-Schiff’s (PAS) reaction as described [22]. Evaluation was conducted in a blinded fashion based on the following criteria: cell swelling, loss of tubular brush border, tubular cell degeneration, sloughing of epithelial cell, and casts in lumen. All parameters were graded on a scale of 0-no lesion, 1-minimal change, 2-mild change, and, 3-prominent change. Finally, comparisons were made between the groups (cold storage group and cold storage +100 µM MitoQ group). All images were taken using a Nikon Eclipse E800 microscope (Q Capture imaging and Nikons Elements software).

### Immunohistochemistry

Immunohistochemical analysis was done as described previously [22]. The primary antibodies against anti-nitrotyrosine (1∶6000 dilution; Millipore, MA, USA) was prepared in antibody diluent solution (0.5% non-fat dry milk and 1% BSA in TBS) and incubated overnight at 4°C. Immunoreactivity was detected by Dako Envision+ System-HRP (Dako, CA, USA). Counterstaining was performed using Mayer’s Hematoxylin (Electron Microscopy Science, PA, USA). Semi quantitative evaluation on nitrotyrosine staining was performed as described [22].

### TUNEL Assay

For visualization of apoptotic cells *in situ* terminal transferase-mediated dUTP nick-end labeling (TUNEL) method was utilized according to the protocol provided by the manufacturer (TACS™ TdT Kit, R&D Systems, MN, USA). Counterstaining was performed using methyl green solution.

### High Resolution Respirometry (HRR)

Complex activity of the electron transport chain (ETC) was measured by high resolution respirometry (HRR) with the OROBOROS Oxygraph-2k (Oroboros instruments, Innsbruck, Austria) according to substrate-inhibitor-titration (SIT) protocol. Briefly, renal biopsies representing cortex and medulla were taken transversely using Speed Cut Biopsy Needle (18G x 10 cm, Gallini). The biopsy specimen was then minced, weighed (6–8 mg, wet weight), and permeabilized with 100 µg/ml saponin prepared in mitochondrial respiration medium MiRO5 [23], [24] by shaking gently at 4°C for 30 min. Permeabilized renal biopsies were then washed 3 times (2 min each) with MiRO5 medium and data acquisition was performed at 37°C. Mitochondrial respiration was initiated by adding 2 mM malate and 10 mM glutamate (Complex I substrate) and maximum active respiration was achieved by adding 2.5 mM ADP. Rotenone (0.2 mM) was then added to completely inhibit complex I respiration. To measure Complex II+III respiration 10 mM succinate (complex II substrate) was added followed by 10 µM antimycin A to inhibit complex III respiration. Finally, Complex IV respiration was monitored by adding 1 mM *N*,*N*,*N*′,*N*′-Tetramethyl-*p*-phenylenediamine (TMPD, substrate for complex IV) made in 0.8 M Ascorbate (pH = 6.0). Inhibition of complex IV was achieved by titrating 800 mM Sodium azide. Finally, data analysis was done using DATLAB 4.2 software (Oroboros) and tissue respiration was presented as oxygen flux (pmol/mg/s).

### Statistical Analysis

Results are presented as mean ± standard error of the mean (SEM). Student's *t* test (paired) was used to compare differences between the mean of paired groups at 95% level of confidence. Differences with a *P* value less than 0.05 were considered statistically significant.

## Results and Discussion

Recently, we have shown that cold storage of rat kidneys leads to increased tubular damage [20], [25] and MitoQ partially protected the cold stored rat kidneys from such damage [20]. The current study was designed for preclinical translation to a large animal renal model to determine whether or not similar protective effects of MitoQ would be observed during cold storage. Preliminary experiments using histopathology (PAS staining) as the primary endpoint for 25, 50, 100 and 200 µM MitoQ doses were tested in at least four kidneys cold stored for 48 hr. The 100 µM MitoQ dose demonstrated the greatest cortical protection (data not shown) and was used in further experiments.

It should be noted that the concentration of MitoQ used in these cold storage studies is quite high compared to other studies, which use nM concentrations. However, since MitoQ uptake within mitochondria is dependent on mitochondrial membrane potential, which is dramatically lowered during cold storage [10]; it is expected that only a fraction of the dose used is actually taken up in renal mitochondria. In addition, the use of intact, whole kidneys also likely impairs the diffusion of MitoQ.

**Figure 1:**
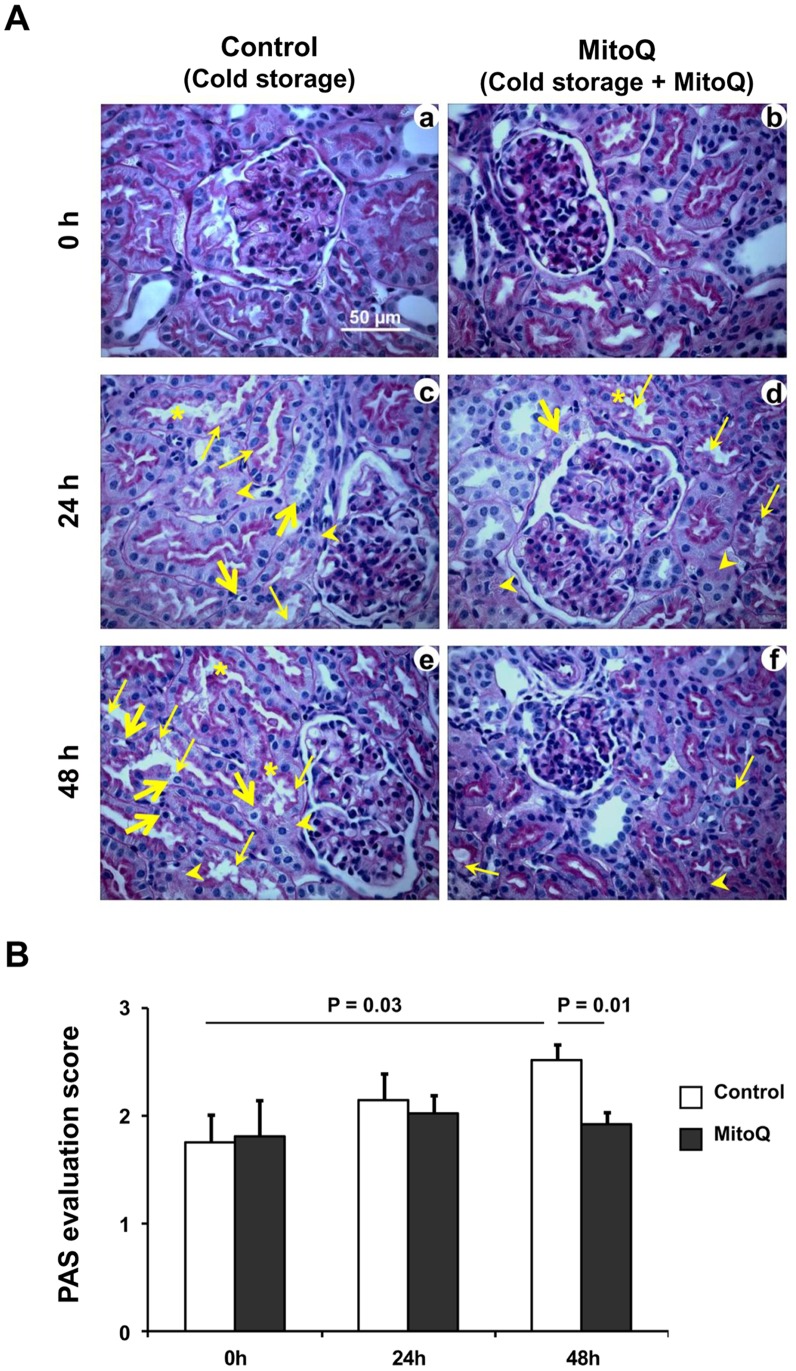
MitoQ (100 µM) blunts tubular injury following cold storage. (A) Representative 400X micrographs of PAS staining in renal cortical section of pig after 24 and 48 h of cold storage (CS) and CS + MitoQ. Representative bar indicates 50 µm. Thin arrows indicate loss of brush border; thick arrows indicate renal epithelial cell detachment and/or cell sloughing; arrow heads indicate epithelial cell degeneration; and asterisks indicate tubular casts. (B) Pathological scoring for tubular injury as a result of cold storage. Error bar indicates Mean ± S.E.M. (n = 5 for both groups). Cold storage significantly induced renal tubular damage at 48 h time point (P = 0.03), and MitoQ significantly blunted this injury (P = 0.01).

The porcine kidney model was selected because canine kidneys in contrast to human are relatively resistant to cold ischemic injury [26]. Primates were not considered due to cost. The porcine kidney is more sensitive to cold preservation [27] and is therefore more likely to demonstrate a clear impact of ischemic injury. The pig was also chosen because its renal anatomy and physiology resemble humans [21]. Although hypothermia reduces the metabolic activity and energy demand [7], renal structural changes occur during static cold preservation [20], [25]. Cold ischemia induced prominent tubular damage including epithelial cell degeneration (arrow heads) loss of brush border (thin arrows), detachment of epithelial cells from basement membrane and sloughing of epithelial cells (thick arrows), and cast formation (asterisks) were observed after 24 and 48 h in the kidneys when compared to the baseline tubular injury of control porcine kidneys (**Fig. 1A** **panel a, 0** **h; panel c, 24** **h; panel e, 48** **h**). Mito Q reduced this cold storage mediated tubular damage after 48 h (**Fig. 1A** **panel b, 0** **h; panel d, 24** **h; panel f, 48** **h**). Semi-quantitative evaluation based on the histopathology review scores showed that renal tubular damage increased significantly after 48 h of cold storage (P = 0.03) compared to baseline, and MitoQ significantly blunted this cold storage mediated tubular damage when compared to the contralateral control cold stored kidneys without MitoQ (**Fig. 1B, P** **= 0.01**). Cold ischemia induces renal tubular cell necrosis and the injury increases with time [28]. Acute tubular necrosis, especially in the papilla/inner medulla, is a common event following transplantation of cold stored renal allografts. We also observed a similar necrotic episode of renal tubules after cold ischemia and surprisingly, MitoQ failed to blunt this tubular necrosis in the inner medulla (**Figure S1**). This could be due to limited availability of MitoQ to this deeper region during flushing and static cold preservation. These results suggest that MitoQ facilitated preservation of the renal tubular structure in the cortex and outer medullary regions in cold storage solution for up to 48 h.

Limited availability of MitoQ in the inner medulla during flush cold preservation could be overcome by hypothermic machine perfusion (HMP) preservation. Significant clinical improvements in the quality of kidney preservation and patient treatment have been reported using HMP [29]–[31]. The results of an international randomized, controlled trial in which hypothermic machine perfusion was compared with static cold stored kidneys demonstrated significantly reduced delayed graft function and improved graft survival when one kidney from each donor was randomly assigned to machine perfusion, and the contralateral organ was assigned to cold storage [29]. More recently, the authors reported three year follow-up data showing survival was better for machine-perfused kidneys (P<0.04) and the survival advantage after machine perfusion was most pronounced from expanded criteria (ECD) donors (86 v 76%, p<0.04) [32]. Future experiments are planned to investigate whether the addition of MitoQ to HMP solution improves preservation of the inner medulla.

**Figure 2:**
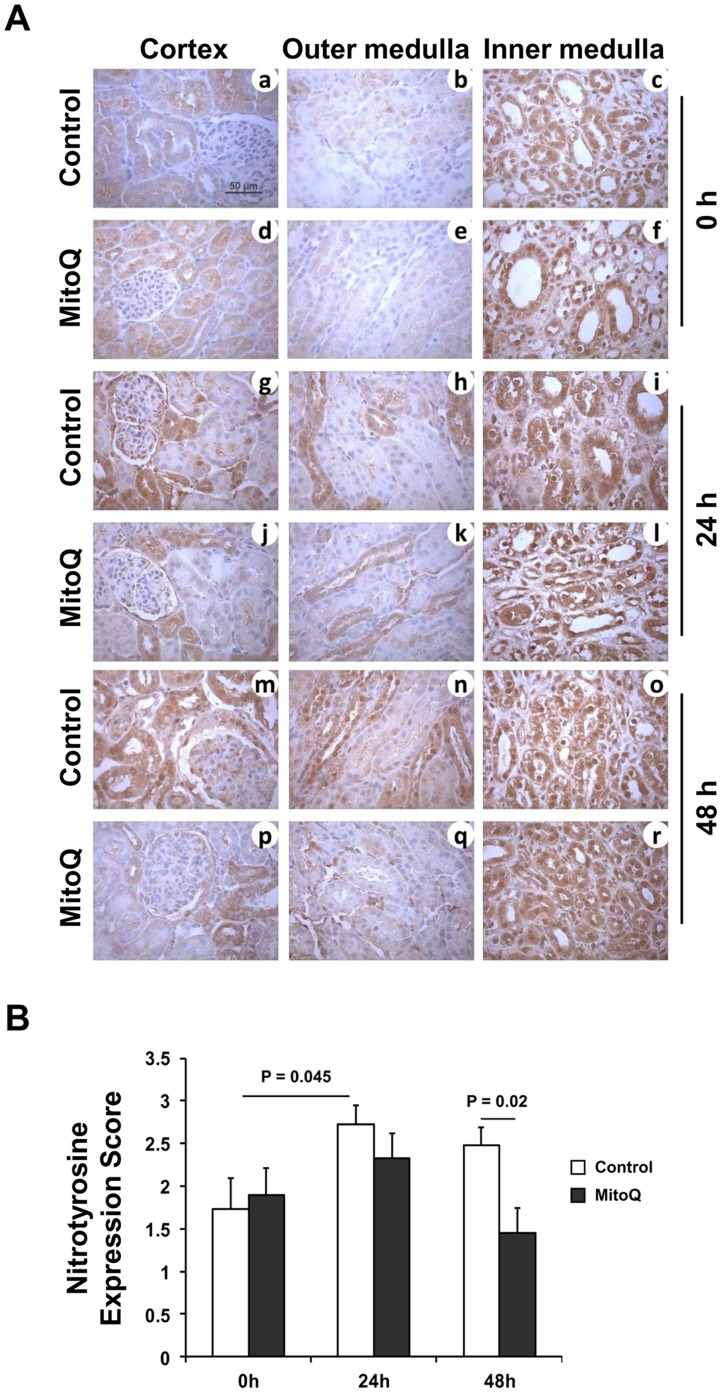
MitoQ blunts protein nitration from oxidative stress during cold storage. (A) Representative 400 X micrographs of nitrotyrosine immunostaining are shown. Representative bar indicates 50 µm. (B) Expression level of Nitrotyrosine was evaluated semi-quantitatively and scored. Error bar indicates Mean ± S.E.M. (n = 5 for both groups). Cold storage significantly increased nitrotyrosine protein accumulation at 24 h time point (P = 0.045). MitoQ significantly blunted protein nitration at 48 h time point (P = 0.02).

Oxidative stress has been shown to play a detrimental role during cold preservation [11], [25], [33], and mitochondria are a potential source of reactive oxygen species (ROS) during cold storage [10], [20]. MitoQ targets mitochondria and scavenges mitochondrial ROS thereby reducing the oxidative stress load [14], [15], [17]–[19], [34], [35]. Using nitrotyrosine as an oxidative stress marker, we evaluated MitoQ’s role in blunting cold ischemia induced oxidative stress in porcine kidneys. In control porcine kidneys exposed to cold storage, nitrotyrosine was present, especially within the inner medullary region (**Fig. 2A**) suggesting that oxidative stress increases rapidly following preservation [36]. Similar to earlier reports in rodent models [20], [25], porcine kidneys also showed increased nitrotyrosine protein accumulation after 24 h and 48 h of cold storage in the cortex and outer medullary region (**Fig. 2A**). As expected, MitoQ blunted cold ischemia induced oxidative stress in the renal tubules after 24 and 48 h as evident by less nitrotyrosine protein accumulation in the cortical and outer medullary region. Surprisingly, nitrotyrosine protein expression in the inner medulla remained unchanged even after addition of MitoQ in the cold storage solution. The reason can be two-fold: 1) the inner medulla actually endured the maximum level of oxidative stress before the organ collection was initiated and MitoQ could not correct the irreversible nature of nitrotyrosine formation; and 2) the concentration of MitoQ during cold storage may be different in the cortex, outer medulla and inner medulla, with the inner medulla concentration being the lowest likely due to poor perfusion during the initial cold flush. Semi-quantitative evaluation based on nitrotyrosine expression score (see methods) showed a significant reduction of nitrotyrosine protein following inclusion of MitoQ at 48 h of cold storage of kidneys when compared to the cold stored kidneys without MitoQ (**Fig. 2B, P** **= 0.02**). These results suggest that MitoQ reduces the oxidative stress load when the organs are stored for up to 48 h.

**Figure 3:**
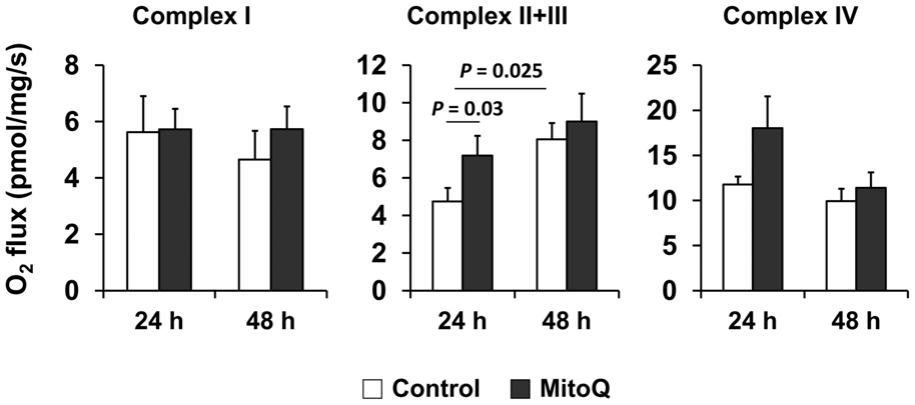
MitoQ improves electron transport chain function during cold storage. Respiration status of complex I, II+III, and IV of the ETC evaluated using SIT protocol (see methods). The graph shows mitochondrial complex respiration (measured as oxygen flux) of fresh renal biopsies that were cold stored with or without MitoQ. Error bar indicates Mean ± S.E.M. (n = 5 for both groups). MitoQ significantly improved complex II+III respiration at 24 h time point (P = 0.03). Cold preservation (without MitoQ) also improved complex II+III respiration after 48 h (P = 0.025).

Previous studies show that cold storage induced mitochondrial oxidative stress leads to alteration of renal ETC activity [10], [20], [25]. Therefore, in this pig model, we studied whether MitoQ altered mitochondrial respiration function during cold storage. Because isolating mitochondria from injured tissues for conventional respiration studies may yield inconsistent populations of mitochondria, we assessed mitochondrial respiration on kidney biopsies using High Resolution Respirometry (HRR). Fresh biopsy specimens from 24 and 48 h cold stored kidneys (with or without MitoQ) were employed to study ETC complexes (I, II+III and IV) using substrate-inhibitor-titration (SIT) protocol (see methods). MitoQ treatment lead to increased complex II+III respiration when compared to the control kidneys cold stored for 24 h (**Fig. 3** **, P = 0.032, 24 h**). This is important since complex III has been shown to be one of the major sites of superoxide generation in cells [25], [37]–[41]. The other complexes (I and IV) respiration remained unaltered by MitoQ addition during cold storage (**Fig. 3** **).**

Interestingly, control kidneys exposed to prolonged cold storage (48 h) resulted in significantly increased mitochondrial complex II+III respiration when compared to the 24 h time point (**Fig. 3** **. P = 0.025**). Since 48 h cold storage increased complex II+III respiration, no further increase was observed with MitoQ treatment at this later time point. The precise mechanism for improved mitochondrial function by prolonged cold storage and MitoQ remains unknown. One possibility is that prolonged cold storage stimulates other compensatory pathways such as autophagy to clear damaged cellular constituents [42], which could include dysfunctional mitochondria (called mitophagy). In addition, it has been shown that cold acclimation [43], [44] and oxidative stress [45]–[47] can induce mitochondrial biogenesis. Increased autophagy and biogenesis could collectively remove damaged mitochondria and restore new mitochondria, hence improve mitochondrial function. In fact, we have rodent data showing that increased mitochondrial superoxide (as a result of MnSOD knockdown) results in increased autophagy and biogenesis (unpublished results). Our new data (porcine) suggest that cold storage (24 hr) induces oxidant production and mitochondrial damage early on (24 hr) and this in turn leads to renal damage (48 hr). Since MitoQ has been shown to modulate mitochondrial ROS formation [14], [15], [17]–[19], [35], it is plausible that reduced ROS leads to improved complex II/III activity. Our data also suggest use of the HRR technique could be quite useful in the determination of donor status prior to transplantation.

**Figure 4:**
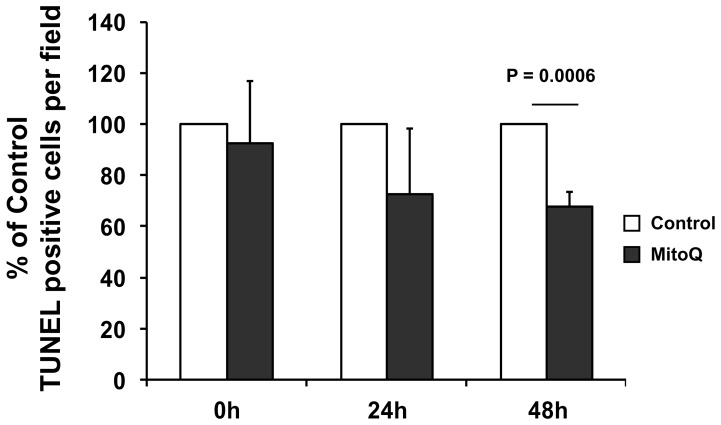
MitoQ blocks cold storage induced renal cell death. TUNEL positive cells were counted in 8 different fields (200X), and the average was reported. The graph shows percentage change of TUNEL positive cells after MitoQ treatment when compared to respective contra lateral control kidneys. Error bar indicates Mean ± S.E.M. (n = 5 for both groups). MitoQ significantly reduced TUNEL positive nuclei after 48 h of cold storage (P = 0.0006).

Cold ischemia causes a rapid depletion of ATP and accumulation of ROS that leads to mitochondrial injury and renal cell death [7]–[10], [25], [33], [48]. We further evaluated whether MitoQ also blocked cold ischemia induced renal cell death in the pig model. Similar to the PAS and nitrotyrosine data, TUNEL staining showed a significant increase of TUNEL positive cells, i.e., renal cell death, after 48 h of cold storage. Also similar to the reduced amount of tubular damage as observed in PAS staining, and decreased oxidative stress observed in nitrotyrosine staining, MitoQ significantly blocked cold storage mediated renal cell death after 48 h, as evident by less TUNEL positive cells in the porcine kidneys (**Fig. 4** **, P = 0.0006**). These results suggest that the antioxidant property of MitoQ may have prevented renal cell death during cold storage. All results combined, indicate that MitoQ partially improved ETC function which blunted cold storage induced oxidative stress, renal tubular damage, and cell death. MitoQ has also been shown to protect against complex I inactivation during hepatic I/R [18] and complex IV inactivation during cisplatin nephrotoxicity [14]. Lu et al. also showed that a functional electron transport chain is not required for MitoQ mediated protection [49]. We believe that MitoQ is working via decreased mitochondrial superoxide production which also reduces downstream ROS production including hydrogen peroxide and peroxynitrite.

The availability of human kidneys for clinical transplantation is limited and the need of transplantable, good quality human organs is growing. It is therefore crucial to improve organ preservation quality to maximize procured renal allograft for clinical transplantation. In this report we demonstrated the significantly improved quality of cadaveric porcine kidneys by addition of 100 µM MitoQ in Belzer’s solution during cold storage (4°C). The addition of MitoQ reduced oxidative stress during cold storage for up to 48 h, which decreased tubular damage, improved mitochondrial function, and led to decreased renal cell death. These results highlight MitoQ as a promising anti-oxidant drug to be included in cold solution (UW) for improved quality and prolonged renal allograft preservation.

## Supporting Information

Figure S1 **MitoQ (100 µM) blunts tubular injury following cold storage.** Representative 400X micrographs of PAS staining in renal cortex, inner and outer medulla of pig after 24 and 48 h of control (cold storage) and MitoQ (cold storage + MitoQ). Representative bar indicates 50 µm. N = 5 for both groups.
(TIF)
