# Supplementary figures and images for: MitoQ Blunts Mitochondrial and Renal Damage during Cold Preservation of Porcine Kidneys
Source: PLoS One. 2012 Nov 6;7(11):e48590. doi: 10.1371/journal.pone.0048590 (PMC3490900; doi:10.1371/journal.pone.0048590)

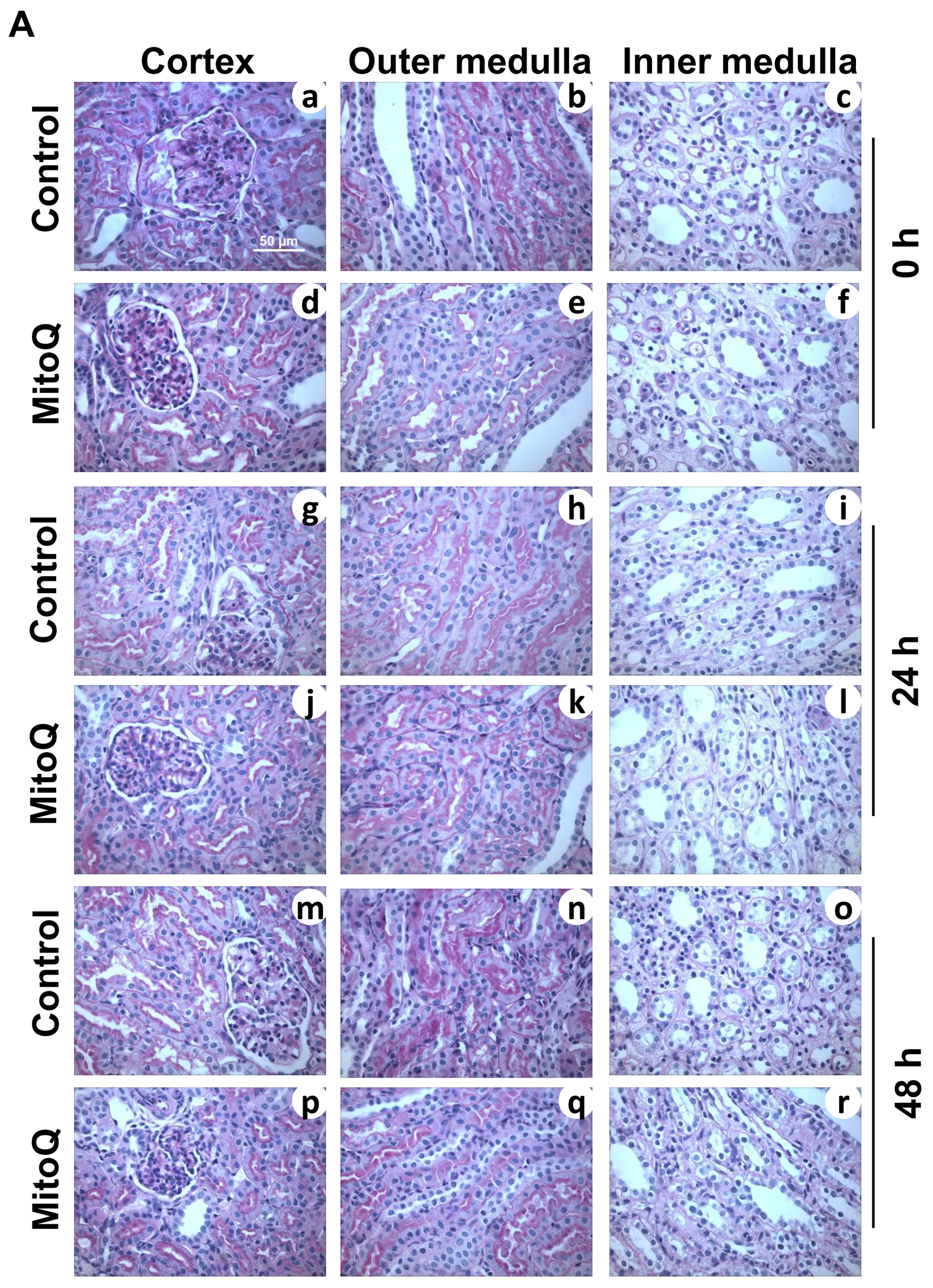

Supplement: Figure S1 — MitoQ (100 µM) blunts tubular injury following cold storage. Representative 400X micrographs of PAS staining in renal cortex, inner and outer medulla of pig after 24 and 48 h of control (cold storage) and MitoQ (cold storage + MitoQ). Representative bar indicates 50 µm. N = 5 for both groups. (TIF) [file pone.0048590.s001.tif]
